# Supplementary material for: The antifungal caspofungin increases fluoroquinolone activity against Staphylococcus aureus biofilms by inhibiting N-acetylglucosamine transferase
Source: Nat Commun. 2016 Nov 3;7:13286. doi: 10.1038/ncomms13286 (PMC5097165; doi:10.1038/ncomms13286)
Supplement: Supplementary Information — Supplementary Figures 1-7, Supplementary Tables 1-2 and Supplementary References [file ncomms13286-s1.pdf]

Supplementary Fig. 1: Concentration-response activity of increasing concentrations of caspofungin against 24 h biofilms from clinical strains and from the reference strain ATCC33591.

24 h biofilms were incubated with increasing concentrations of caspofungin (CAS; 40 to 10240 mg L^{-1}) for 48 h. The ordinate shows the change in resorufin fluorescence (RF; filled symbols and thick lines) or in crystal violet absorbance (CV; open symbols and thin dotted lines) expressed in percentage of the corresponding control value (no caspofungin added; corresponding to CT values on the graphs). All values are means ± SD of 4 replicates. The vertical dotted line corresponds to the concentration of caspofungin (40mg L^{-1}) used in combination with fluoroquinolones in the *in vitro* biofilm models presented in the main part of the paper.

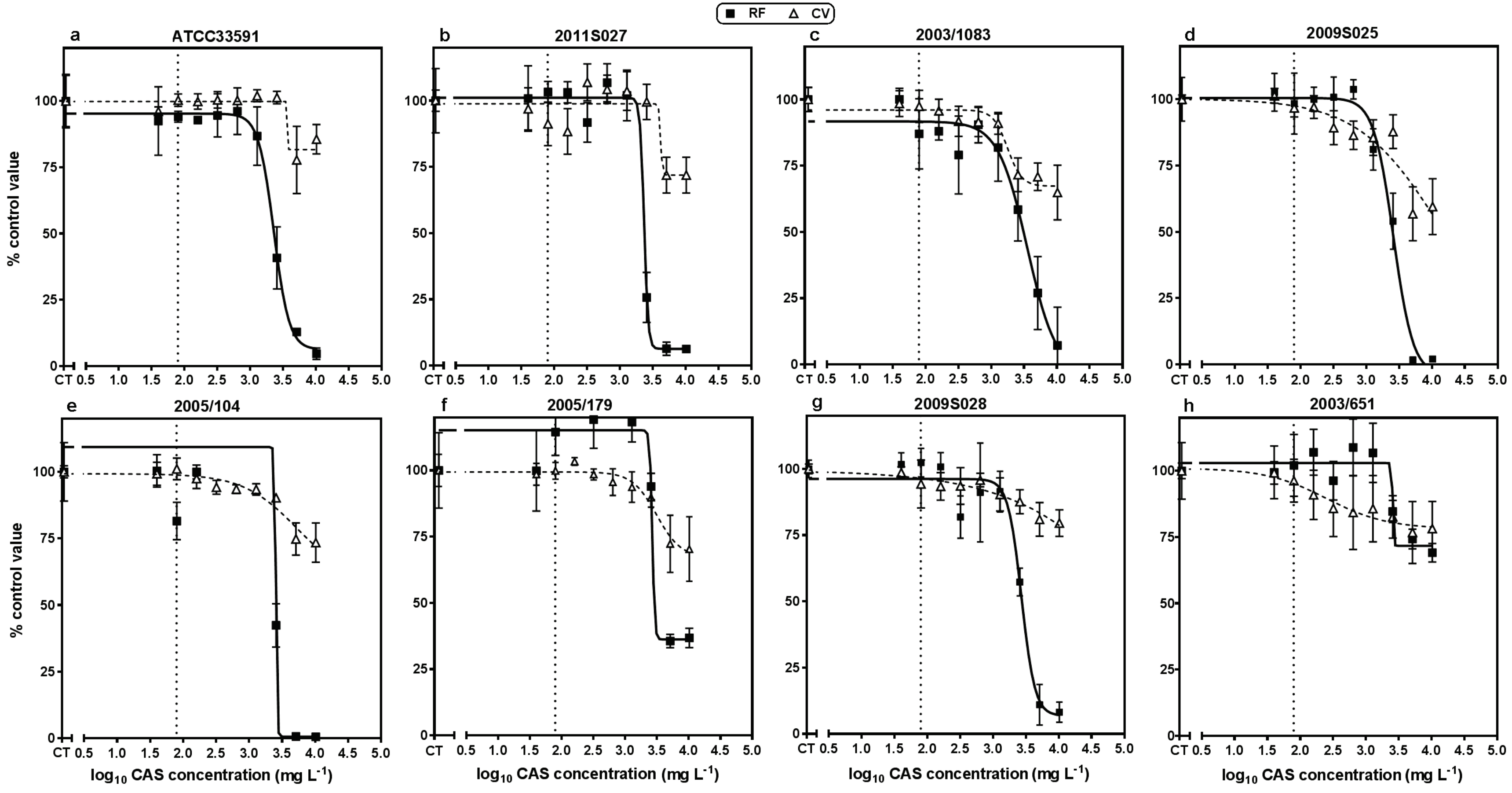

Supplementary Fig. 2: Effect of fluoroquinolones alone or combined with caspofungin on biofilms from 4 selected strains.

Biofilms were incubated during 48 h in the absence or in the presence of the drugs (moxifloxacin [a-h; black] or delafloxacin [i-p; blue] at increasing concentrations and used alone [a-d and i-l] or combined [e-k and m-p] with 40 mg L^{-1} caspofungin). The ordinate shows resorufin fluorescence (closed bars; left scale; expressed in percentage of the value measured in control conditions [no fluoroquinolone added]) and CFU (open bars; right scale; expressed in log_{10} units). Data are the mean ± standard deviation (SD) of 4 replicates. Statistical analysis: multiple t-tests comparing data for fluoroquinolone alone or combined with caspofungin in the same conditions: ***: p < 0.001; **: p < 0.01; * p< 0.05.

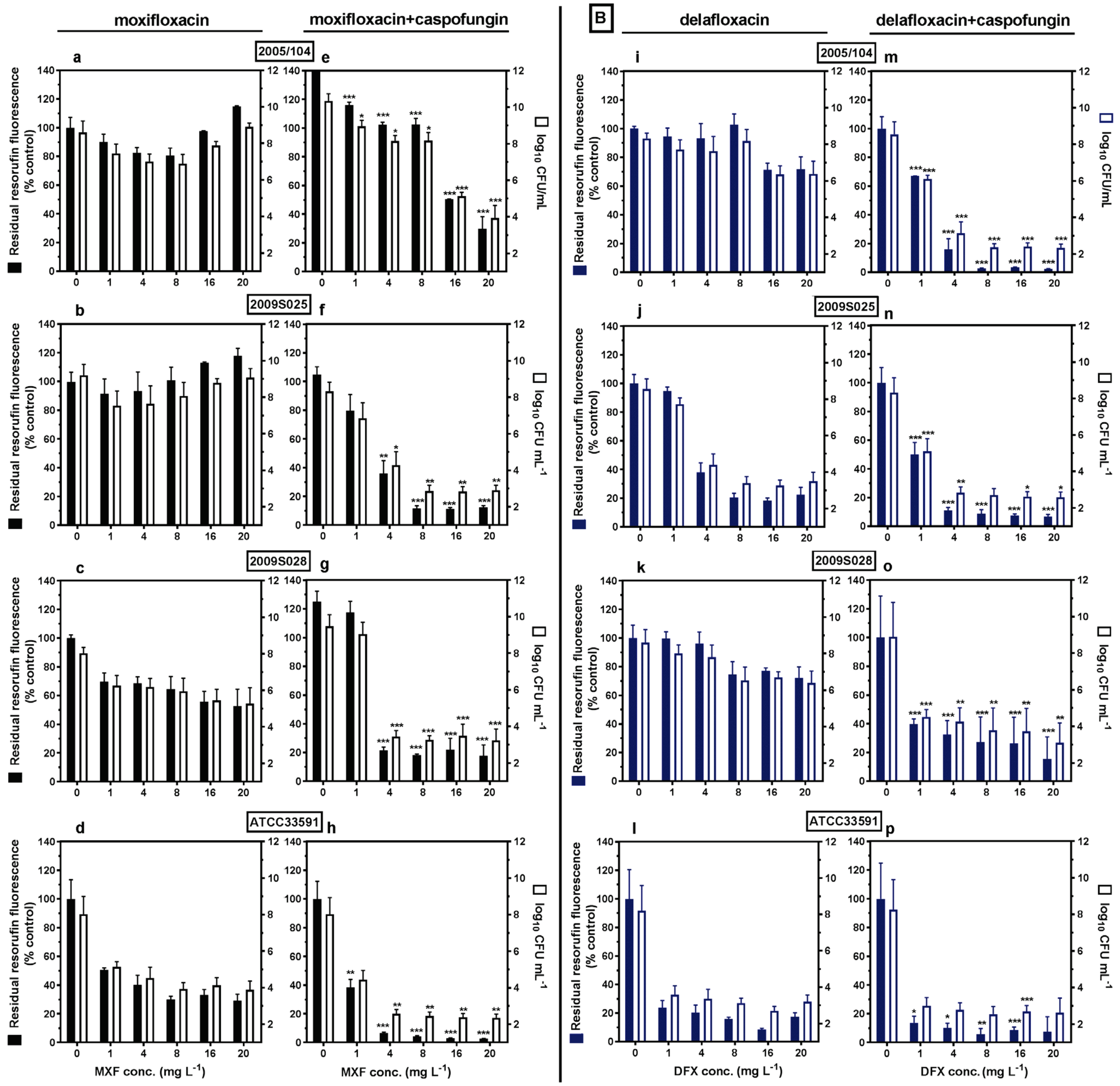

Supplementary Fig. 3: Concentration-response relationships for the activity of fluoroquinolones alone or combined with caspofungin against biofilms from 4 selected strains

Left: moxifloxacin alone (a-d) or combined with 40 mg L^{-1} caspofungin (e-h); right: delafloxacin alone (i-l) or combined with 40 mg L^{-1} caspofungin (m-p). Data are expressed in percentage of control values (no drug added; corresponding to CT values on the graphs) for viability (assessed by measuring the fluorescence of resorufin [RF; closed squares or circles]) or biomass (assessed by crystal violet staining [CV; open triangles]). Data are means ± standard deviation (SD) of 4 replicates.

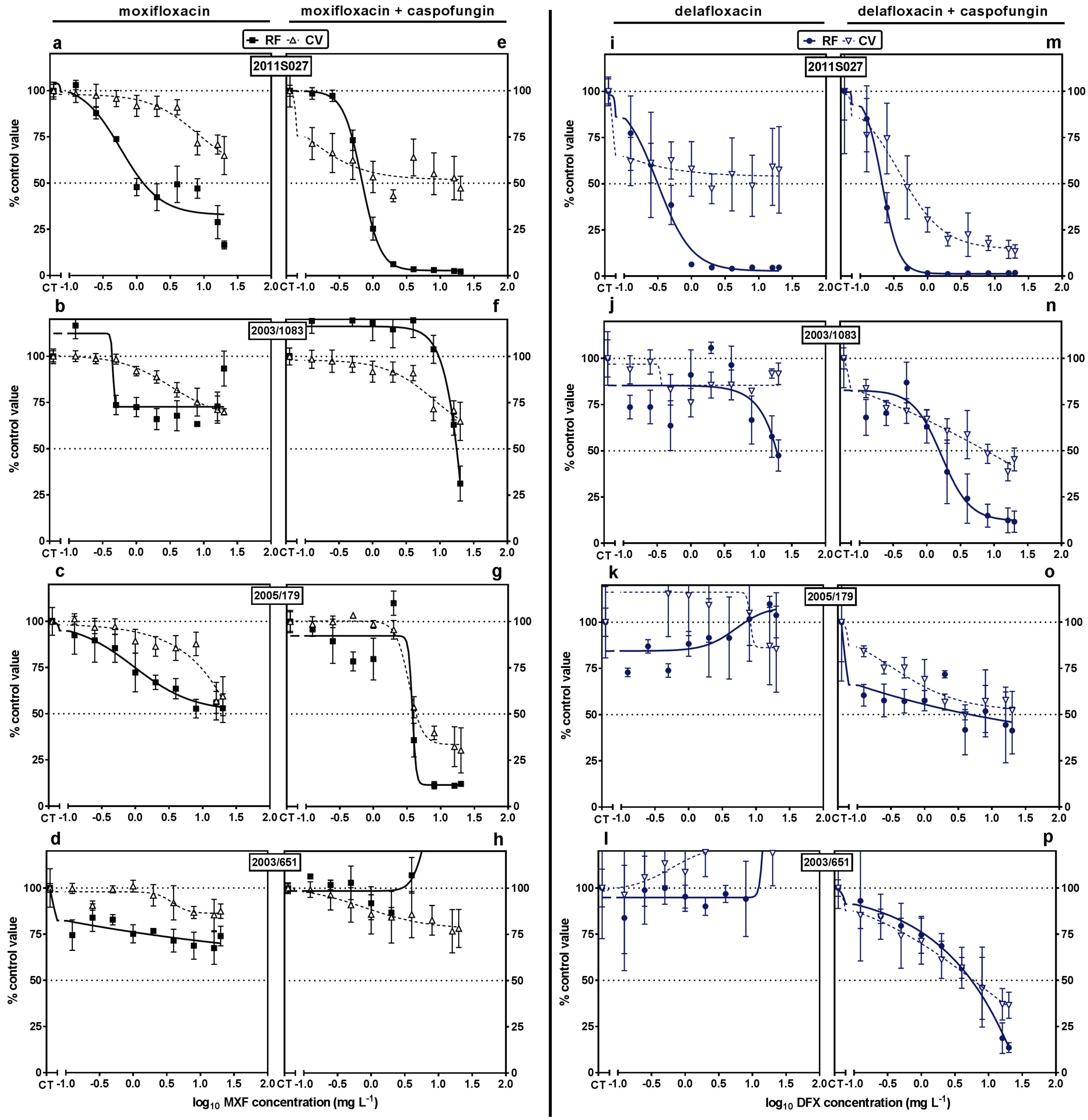

Supplementary Fig. 4: Influence of caspofungin on extracellular DNA and proteins in biofilms from 4 selected strains.

Extracellular DNA (a) and proteins (b) present in biofilms were quantified in biofilms cultivated in control conditions (CTRL) or after 48 h incubation with 40 mg L^{-1} caspofungin (+ CAS). Values are means ± SD of three independent determinations. Statistical analysis: multiple t-tests comparing data for untreated or caspofungin treated biofilms: no significant differences were observed.

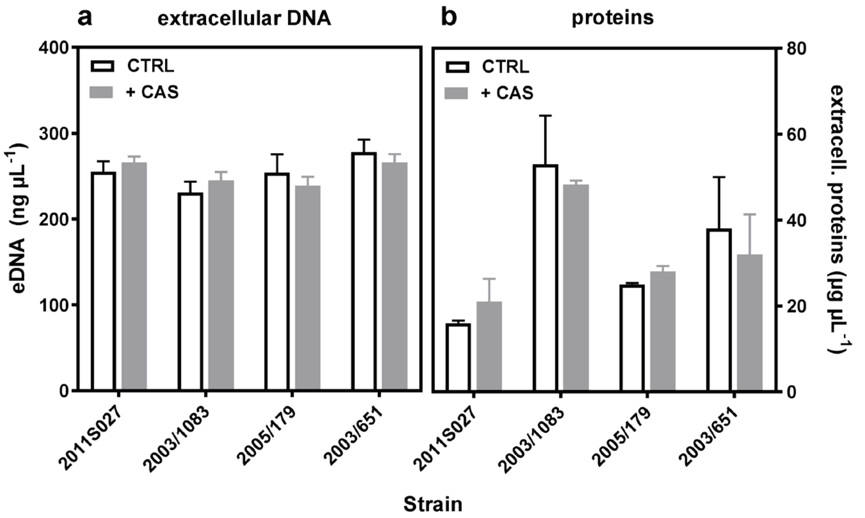

Supplementary Fig. 5: sequence alignment of *S. aureus* icaA and fungal 1,3 glucan synthases.

Partial alignment of protein sequences of *S. aureus* icaA (412 amino acids) and fungal 1,3 glucan synthases from *Candida europaea* (1946aa); *C. yegresii* (1931aa); *Trichophyton rubrum* (1791aa); *Pyrenophora tritici-repentis* (1943 aa); *C. parapsilosis* (1655aa); *C. orthopsilosis* (1586aa); *C. albicans* (1572 aa); *C. dubliniensis* (1571aa); *C.maltosa* (1580 aa); *C. tropicalis* (1572 aa) ; *Scheffersomyces stipitis* (1695 aa). The regions containing the amino acids that are predicted to function as catalytic residues (Asp^{134}; Asp^{227} and Arg^{276}) in IcaA are conserved between *S. aureus N*-acetyl-glucosamine transferase and fungal 1,3 β-glucan synthases (the conserved amino acids are indicated by X).

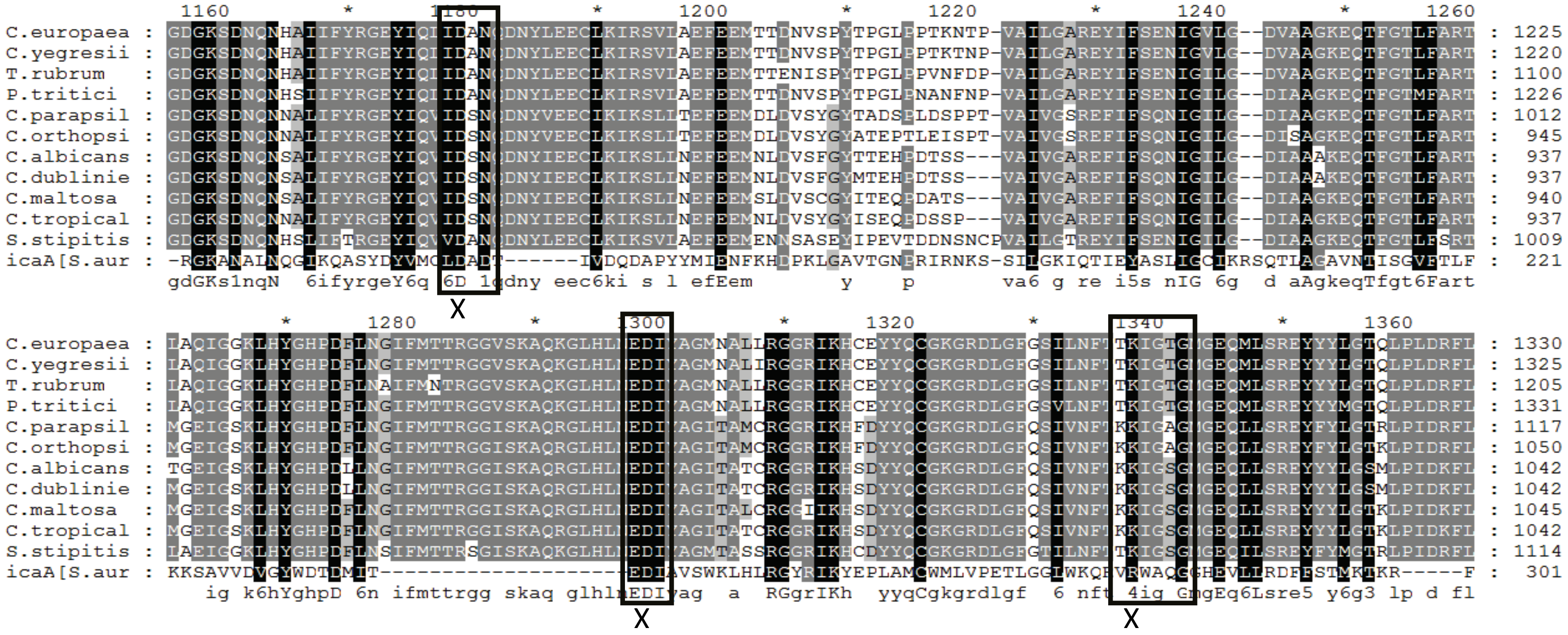

Supplementary Fig. 6: Sequence comparison of *icaA* gene between ATCC33591 and 2003/651

Alignment of DNA sequences of ATCC33591 *icaA* and 2003/651 *icaA* with the previously published sequence of *icaA* (database accession no AIW28333) was performed using GeneDoc software. No substitutions were observed in ATCC33591 *icaA*. In the 2003/651 isolate, six base-pair substitutions were noticed (positions in gray): $T_{272}C$; $A_{416}G$; $T_{479}C$; $T_{536}C$; $A_{635}G$; $A_{1088}G$. All of these substitutions corresponded to silent mutations (no modification in the amino acids produced).

```
                   220         *        240         *        260         *        280         *        300         *         3
2003/651  : TTTATCCTGTATTTATGTCTATTTACTGGATTGTCGGTTCAATTTATTTCTATTTTACCAGAGAAATTAGATATTCATTGAACAAGAAGCCTGACATAAATGTGGA :  318
ATCC33591 : TTTATCCTGTATTTATGTCTATTTACTGGATTGTCGGTTCAATTTATTTCTATTTTACTAGAGAAATTAGATATTCATTGAACAAGAAGCCTGACATAAATGTGGA :  318
icaA      : TTTATCCTGTATTTATGTCTATTTACTGGATTGTCGGTTCAATTTATTTCTATTTTACTAGAGAAATTAGATATTCATTGAACAAGAAGCCTGACATAAATGTGGA :  318
            TTTATCCTGTATTTATGTCTATTTACTGGATTGTCGGTTCAATTTATTTCTATTTTACtAGAGAAATTAGATATTCATTGAACAAGAAGCCTGACATAAATGTGGA

            20         *        340         *        360         *        380         *        400         *        420
2003/651  : TGAATTAGAAGGCATTACATTTTTACTTGCCTGTTATAACGAAAGTGAAACGATTGAAGATACGTTGTCTAATGTTCTTGCACTCAAATACGAGAAGAAAGAAATT :  424
ATCC33591 : TGAATTAGAAGGCATTACATTTTTACTTGCCTGTTATAACGAAAGTGAAACGATTGAAGATACGTTGTCTAATGTTCTTGCACTCAAATACGAGAAAAAAGAAATT :  424
icaA      : TGAATTAGAAGGCATTACATTTTTACTTGCCTGTTATAACGAAAGTGAAACGATTGAAGATACGTTGTCTAATGTTCTTGCACTCAAATACGAGAAAAAAGAAATT :  424
            TGAATTAGAAGGCATTACATTTTTACTTGCCTGTTATAACGAAAGTGAAACGATTGAAGATACGTTGTCTAATGTTCTTGCACTCAAATACGAGAAaAAAGAAATT

                 *        440         *        460         *        480         *        500         *        520         *
2003/651  : ATTATCATTAATGATGGAAGTTCAGATAATACAGCAGAACTCATCTATAAAATCAAAGAAAATAATGACTTTATTTTCGTCGATTTACAAGAAAACAGAGGTAAAG :  530
ATCC33591 : ATTATCATTAATGATGGAAGTTCAGATAATACAGCAGAACTCATCTATAAAATTAAAGAAAATAATGACTTTATTTTCGTCGATTTACAAGAAAACAGAGGTAAAG :  530
icaA      : ATTATCATTAATGATGGAAGTTCAGATAATACAGCAGAACTCATCTATAAAATTAAAGAAAATAATGACTTTATTTTCGTCGATTTACAAGAAAACAGAGGTAAAG :  530
            ATTATCATTAATGATGGAAGTTCAGATAATACAGCAGAACTCATCTATAAAATtAAAGAAAATAATGACTTTATTTTCGTCGATTTACAAGAAAACAGAGGTAAAG

                   540         *        560         *        580         *        600         *        620         *
2003/651  : CCAACGCACTCAATCAAGGCATTAAACAGGCTTCATATGATTATGTAATGTGCTTGGATGCAGATACTATCGTTGATCAAGATGCACCATATTATATGATTGAGAA :  636
ATCC33591 : CCAATGCACTCAATCAAGGCATTAAACAGGCTTCATATGATTATGTAATGTGCTTGGATGCAGATACTATCGTTGATCAAGATGCACCATATTATATGATTGAAAA :  636
icaA      : CCAATGCACTCAATCAAGGCATTAAACAGGCTTCATATGATTATGTAATGTGCTTGGATGCAGATACTATCGTTGATCAAGATGCACCATATTATATGATTGAAAA :  636
            CCAAtGCACTCAATCAAGGCATTAAACAGGCTTCATATGATTATGTAATGTGCTTGGATGCAGATACTATCGTTGATCAAGATGCACCATATTATATGATTGAaAA

                      *       1080         *       1100         *       1120         *       1140         *       1160
2003/651  : ATTTTGATGTTTGAGCAAATCATCTCGATTTTATGGGTATATATAGTGCTTCTATATTTAGGCTATTTGTTCATAACAGCAAACTTCTTAGACTATACATTTATGA : 1166
ATCC33591 : ATTTTGATGTTTGAGCAAATCATCTCAATTTTATGGGTATATATAGTGCTTCTATATTTAGGCTATTTGTTCATAACAGCAAACTTCTTAGACTATACATTTATGA : 1166
icaA      : ATTTTGATGTTTGAGCAAATCATCTCAATTTTATGGGTATATATAGTGCTTCTATATTTAGGCTATTTGTTCATAACAGCAAACTTCTTAGACTATACATTTATGA : 1166
            ATTTTGATGTTTGAGCAAATCATCTCaATTTTATGGGTATATATAGTGCTTCTATATTTAGGCTATTTGTTCATAACAGCAAACTTCTTAGACTATACATTTATGA
```

Supplementary Fig. 7: calibration curve used for the determination of UDP concentration.

Increasing concentrations of UDP (0.005-50 µM) were prepared in reaction buffer (250 mM MES-NaOH buffer, 40 mM UDP-GlcNAc, 20 mM $MnCl_2$, 400 mM *N*-acetylglucosamine, 1% (w/v) Triton X-100, pH 6.25). Five µL of UDP detection mixture containing 4.5 µg mL^{-1} of UDP antibody (Transcreener UDP^2 kit) were added to the samples. The Fluorescence Polarization response is given as mP (*milli*-Polarization level)[1]; see equation of the calibration curve below the graph.

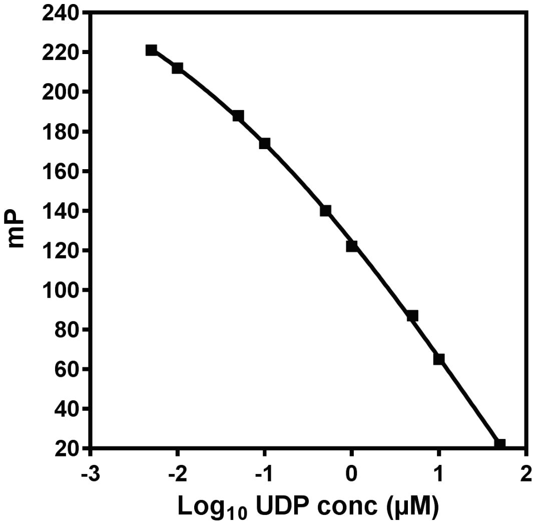

mP: $-237.5+(530.79/1+10^{(-0.2054\ [1.604-\log \text{UDP conc}])})$

$R^2 = 0.9996$

Supplementary Table 1: **relative potencies of antibiotics alone or combined with caspofungin against biofilms *in vitro.***

Antibiotic concentrations needed to achieve 50 % reduction in bacterial viability, as calculated from the Hill equation of the concentration-effect curve for experiments similar to those described in Supplementary Fig. 3, for 24-h biofilm exposed during 48 h to antibiotics alone or combined with 40 mg L^{-1} caspofungin (CAS). Data for moxifloxacin (presented in Table 1) are reproduced here for sake of comparison. Values in bold denote an increased activity of the antibiotic in the presence of caspofungin.

	**Antibiotic concentrations (mg L^{-1})**							
	**Moxifloxacin (MXF)**		**Daptomycin (DAP)**		**Vancomycin (VAN)**		**Linezolid (LDZ)**	
strains	MXF	MXF+CAS	DAP	DAP+CAS	VAN	VAN+CAS	LDZ	LDZ+CAS
2003/1083	>20 [b]	**17**	>20	>20	>20	>20	>20	**15.9**
2009S025	>20	**1.9**	>20	>20	>20	>20	>20	>20
2011S027	0.9	**0.7**	15.4	**0.8**	>20	>20	9.8	**3.5**
2003/651	>20	>20	>20	>20	>20	>20	>20	>20
2005/104	>20	**18**	>20	>20	>20	>20	>20	**18.4**
2005/179	>20	**3.8**	>20	>20	>20	>20	>20	>20
2009S028	>20	**3.7**	>20	>20	>20	>20	>20	>20
ATCC33591	1.25	**0.1**	>20	>20	>20	>20	17.7	**1.8**

[a] Reduction in signal as compared to untreated biofilm

[b] >20: no decrease in signal at a concentration of 20 mg L^{-1} in antibiotic

Supplementary Table 2: *icaABCD* mRNA levels in biofilms of reference and clinical isolates.

**strain**	**mRNA expression levels of *icaABCD* genes in *S. aureus* isolates 24-h biofilms[a]**			
	icaA	***icaB***	***icaC***	***icaD***
ATCC33591	1	1	1	1
2011S027	1.8 ± 0.5*	0.9 ± 0.2	1.2 ± 0.1	5.1 ± 0.1*
2003/1083	4.0 ± 0.6 *	1.0 ± 0.3	1.3 ± 0.1	0.9 ± 0.6
2009S025	2.5 ± 0.5*	5.1 ± 0.4*	0.9 ± 0.2	1.5 ± 0.3
2005/104	4.2 ± 0.4*	0.8 ± 0.1	1.1 ± 0.2	4.9 ± 0.3*
2005/179	6.0 ± 0.9*	5.6 ± 0.3*	1.2 ± 0.5	5.0 ± 0.8*
2009S028	4.1 ± 0.2*	2.8 ± 0.5*	0.9 ± 0.1	1.6 ± 0.7
2003/651	16.3 ± 0.7*	2.8 ± 0.4*	1.5 ± 0.4	4.8 ± 1.0*

[a] transcript levels for clinical isolates relative to those measured in ATCC33591. Data are mean ± SD of triplicates. Statistical analysis: * indicates a significant difference as compared to strain ATCC33591 ($p<0.05$). Data were normalized using 16s RNA gene as housekeeping gene.

All the clinical strains under study show an overexpression of *icaA*, but to variable levels; overexpression of the other genes is not systematic, as previously described[2]. A possible explanation is that the *icaADBC* operon is under the control of different transcription factors like Rbf but also SarX, which binds to *icaA* and specifically regulates the expression of this gene[3].

Supplementary References

(1) Wagner G.K. & Pesnot T. Glycosyltransferases and their assays. *Chembiochem*. **11**, 1939-1949 (2010).

(2) Atshan S.S. *et al*. Quantitative PCR analysis of genes expressed during biofilm development of methicillin resistant *Staphylococcus aureus* (MRSA). *Infect. Genet. Evol*. **18**, 106-112 (2013).

(3) Cue D., Lei M.G. & Lee C.Y. Activation of *sarX* by Rbf is required for biofilm formation and ica*ADBC* expression in *Staphylococcus aureus*. *J. Bacteriol*. **195**, 1515–1524 (2013).
